# Atlas of Skin Diseases

**Published:** 1877-05

**Authors:** 


### BIBLIOGRAPHICAL.

Atlas of Skin Diseases. By Louis A. Duhring, M.D., Prof. of Skin Diseases in the Hospital of the University of Pennsylvania; Physician to the Dispensary for Skin Diseases, of Philadelphia, etc. J. B. Lippincott & Co., Publishers, Philadelphia, 1877. $2.50 each part.

This is the second part of this very valuable work, and in it is portrayed with life-like vividness cases of *acne rosacca icthyosis* (simplex) *tinia versicolar*, and *sycosis non-parasitica.* The publishers propose in this series of practical illustrations to furnish a means by which the more common affections of the skin may be easily recognized by those whose clinical advantages are limited; and judging by the edition before us we think the publication fully merits the patronage of the profession, and will justify the expectation of its projectors.

The series will be completed in eight, or not exceeding ten parts, issued quarterly, each part containing four plates, royal quarto size, with accompanying texts and explanations.

The enterprise is calculated to remove much of doubt and confusion in diagnosis, and thus supplant the too frequent empirical practice by a rational course of treatment in skin affections. We are of the opinion that no one should be without it, who expects to derive much satisfaction in treating such diseases, and therefore heartily recommend it to the profession.

